# Nitrogen deficiency modulates carbon allocation to promote nodule nitrogen fixation capacity in soybean

**DOI:** 10.1002/EXP.20230104

**Published:** 2023-11-30

**Authors:** Xiaolong Ke, Han Xiao, Yaqi Peng, Xue Xia, Xuelu Wang

**Affiliations:** ^1^ State Key Laboratory of Crop Stress Adaptation and Improvement, School of Life Sciences Henan University Zhengzhou China; ^2^ The Academy for Advanced Interdisciplinary Studies Henan University Zhengzhou Henan China; ^3^ Sanya Institute of Henan University Sanya Hainan China; ^4^ School of Environmental and Life Sciences, College of Engineering, Science and Environment University of Newcastle Callaghan New South Wales Australia

**Keywords:** carbon allocation, energy sensor, nitrogen deficiency, nitrogen fixation capacity, soybean, symbiotic nitrogen fixation

## Abstract

Previously, the effect of soil mineral N deficiency on nodule nitrogen fixation capacity (NFC) is unclear. In this study, we found that N deficiency would enhance sucrose allocation to nodules and PEP allocation to bacteroid to promote nodule NFC. Our findings provide new insights into the design of leguminous crops with improved adaptation to fluctuating N levels in the soil.

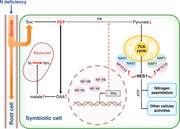

Nitrogen (N) is an essential inorganic nutrient that supports plant growth and development. Plant N acquisition is achieved mainly through the uptake of soil mineral N, while legumes also obtain N through establishing symbiotic relationships with rhizobia. Symbiotic nitrogen fixation (SNF) is a highly energy‐consuming process with a carbon (C) consumption of >5 g C g N^−1^, which costs far more than soil N uptake that generally does not exceed 2.5 g C g N^−1^.^[^ ^1^, ^2^, ^3^ ^]^ Therefore, it is reasonable that sufficient mineral N in the soil would inhibit nodulation and nitrogen fixation, including rhizobial infection, nodule initiation, growth, and nitrogen fixation.^[^ ^4^, ^5^ ^]^ In contrast, N deficiency in the soil may enhance nodulation in legumes. Autoregulation of nodulation (AON) is an important strategy for legumes to control nodule number when sufficient rhizobia infection or mineral N exists.^[^ ^6^, ^7^, ^8^ ^]^ It was recently reported that N deficiency would enhance the expression of C‐terminally encoded peptides (CEPs) in roots to activate the shoot Compact Root Architecture 2 (CRA2) receptor, thereby inhibiting the systemic AON to promote nodule formation in *Medicago truncatula*.^[^ ^9^, ^10^, ^11^ ^]^ However, whether N deficiency also affects nodule nitrogen fixation capacity (NFC) at later stages remains unclear.

To examine the effect of N deficiency on nodule NFC, we used nutrient solution without nitrate for the soybean “Williams 82” (W82) plants inoculated with *Bradyrhizobium diazoefficiens* USDA110 at 10 days post inoculation (DPI). At 25 DPI, as compared with the N‐sufficient plants supplied with 2 mM nitrate according to the previous reports,^[^ ^12^, ^13^, ^14^ ^]^ the soybean plants with deficient N supply showed similar nodule number and weight, but significantly enhanced nodule nitrogenase activity (Figure 1A), suggesting that N deficiency at late stages can strongly promote nodule NFC in soybean. Moreover, at late stages, N deficiency also inhibited shoot growth, but had no effect on root and nodule development (Figure S1).

**FIGURE 1:**
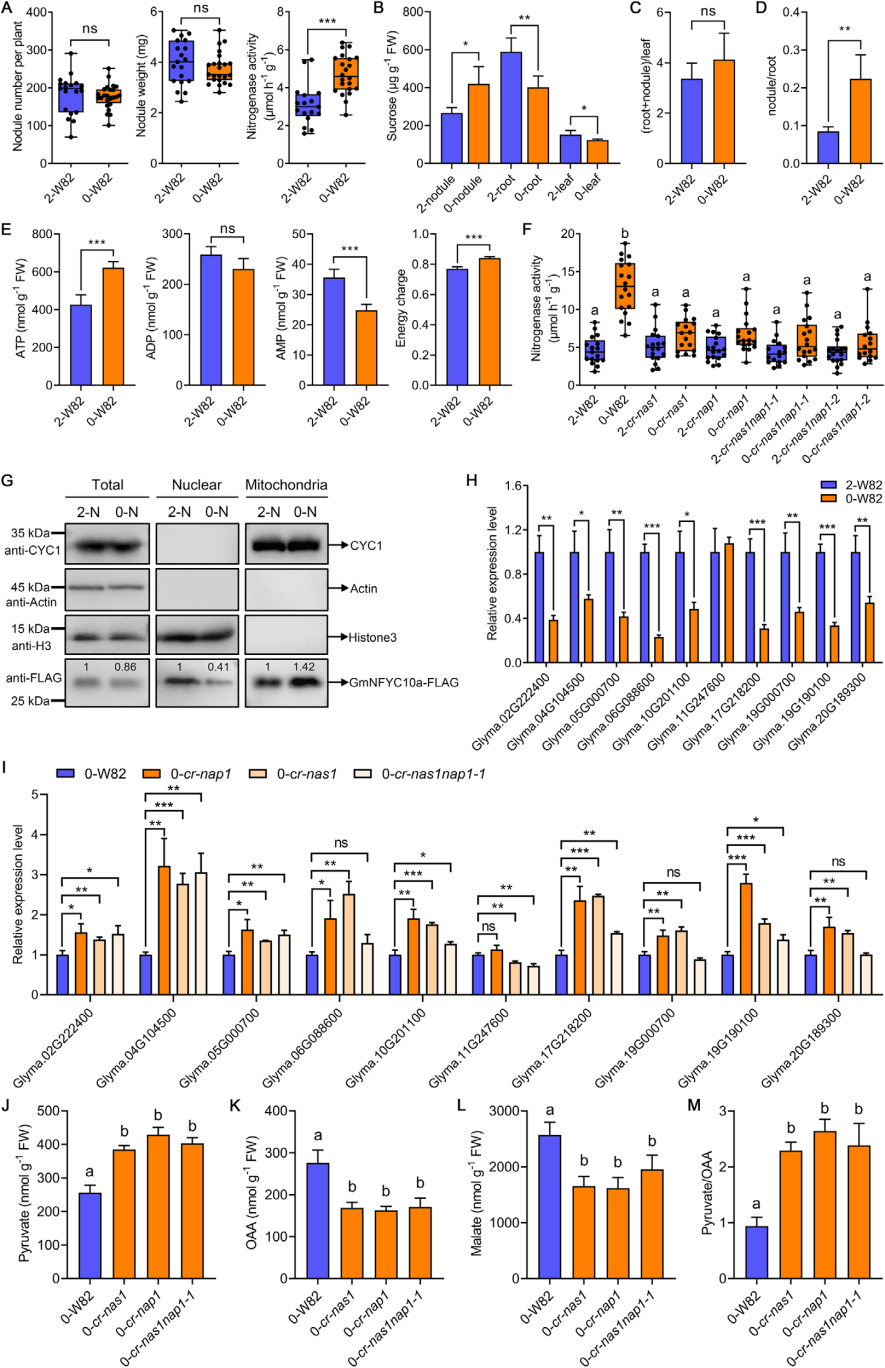
GmNAS1 and GmNAP1 regulate the enhanced nodule NFC under N deficiency. (A) Nodulation assays of W82 plants under different N conditions. 2‐W82, W82 plants supplied with 2 mM KNO_3_; 0‐W82, W82 plants supplied with 0 mM KNO_3_. (B) The sucrose concentration in nodules, roots, and leaves of W82 plants under different N conditions. FW, fresh weight. (C) The sucrose content ratio of underground tissues (including root and nodule) to leaves in 2‐W82 and 0‐W82 plants. (D) The sucrose content ratio of nodule to root in 2‐W82 and 0‐W82 plants. (E) The ATP/ADP/AMP contents and energy charge in nodules of W82 plants under different N conditions. (F) Nodule nitrogenase activity of W82 and GmNAS1/GmNAP1 mutants under different N conditions. (G) GmNFYC10a‐FLAG protein abundance in the nucleus and mitochondria of nodule cells. 2‐N, *gGmNFYC10a‐FLAG* transgenic plants supplied with 2 mM KNO_3_; 0‐N, *gGmNFYC10a‐FLAG* transgenic plants supplied with 0 mM KNO_3_. Ubiquinol‐cytochrome C oxidoreductase subunit CYC1, actin, and histone H3 were used as mitochondrial, cytoplasmic, and nuclear markers, respectively. The numbers above the lanes indicate relative band intensity quantified by ImageJ. (H) Relative expression levels of glycolytic genes in W82 nodules under different N conditions. (I) Relative expression levels of glycolytic genes in W82, *cr‐nap1*, *cr‐nas1*, and *cr‐nas1nap1‐1* nodules under N‐deficiency conditions. Pyruvate (J), OAA (K), and malate (L) contents and ratio of pyruvate to OAA (M) in W82, *cr‐nas1*, *cr‐nap1*, and *cr‐nas1nap1‐1* nodules under N‐deficiency conditions. Data in (B–E) and (J–M) are means of four biological replicates ± standard deviation (SD). Significant differences were determined by Student's *t* test (^*^ *p* < 0.05, ^**^ *p* < 0.01, ^***^ *p* < 0.001) in (A–E), (H), and (I), or one‐way analysis of variance (ANOVA) and post hoc Tukey's test with different lowercase letters indicating significant differences (*p* < 0.05) in (F) and (J–M); ns, not significant.

It was previously reported that N deficiency of the whole plant possibly increases the sucrose content of nodules in *M. truncatula*.^[^ ^15^ ^]^ Here we found that the late N deficiency significantly increased nodule sucrose concentration in soybean (Figure 1B). Accordingly, nodule energy state (NES) was elevated after N deficiency (Figure 1E). We also found that the sucrose concentrations in both roots and leaves were significantly reduced under N‐deficiency conditions (Figure 1B). However, the sucrose content ratio of underground tissues (including roots and nodules) to leaves was only slightly increased by N deficiency (Figure 1C). Remarkably, the sucrose content ratio of nodule to root was greatly increased under N‐deficiency conditions (Figure 1D). The results suggested that the increase in nodule sucrose concentration under N‐deficiency conditions is mainly due to more sucrose allocated to nodules from roots, but not due to more sucrose transported to underground tissues from leaves.

We have previously found that two nodule‐specific mitochondria‐localized energy sensors in soybean, GmNAS1 and GmNAP1, can sense the increased NES to reduce GmNFYC10 nuclear accumulation, thereby modulating glycolytic pathway and phosphoenolpyruvate (PEP) allocation to enhance nodule NFC.^[^ ^16^ ^]^ To investigate whether GmNAS1/GmNAP1 mediates the enhanced nodule NFC under N‐deficiency conditions, we examined the nodulation phenotypes of knockout mutants of *GmNAS1*/*GmNAP1* created in our previous study,^[^ ^16^ ^]^ including *cr‐nas1*, *cr‐nap1*, *cr‐nas1nap1‐1*, and *cr‐nas1nap1‐2*, under different usable nitrogen conditions. The result suggested that the nodule number and weight of these mutants were similar to the wild type under N‐deficiency conditions (Figure S2A,B), and the development of nodule infection zone was also unaffected in these mutants (Figure S2C). However, N deficiency failed to enhance nodule NFC in all of these mutants (Figure 1F), indicating that GmNAS1/GmNAP1 are required for the N‐deficiency‐enhanced nodule NFC. Moreover, N deficiency also failed to enhance nodule NFC in the *cr‐nfyc10* mutant (Figure S3), indicating that *GmNFYC10* is also necessary for the elevated NFC after N deficiency. Together, these results suggested that the GmNAS1/GmNAP1‐GmNFYC10 module regulates the increase in nodule NFC under N‐deficiency conditions.

Previous study showed that the interaction of GmNAS1/GmNAP1 with GmNFYC10a was inhibited by increased AMP concentration but enhanced by high NES.^[^ ^16^ ^]^ Considering that NES was elevated after N deficiency (Figure 1E), we tested and found that the interaction of GmNAS1/GmNAP1 with GmNFYC10a was also enhanced under N‐deficiency conditions (Figure S4A), while the expression levels of *GmNAS1*, *GmNAP1*, and *GmNFYC10a* were unchanged (Figure S4B). Thus, under N‐deficiency conditions, more GmNFYC10a protein was anchored to the mitochondria by GmNAS1/GmNAP1, and less GmNFYC10a protein was localized to the nucleus (Figure 1G). We further observed that the expression of many glycolytic genes activated by GmNFYC10 was downregulated after N deficiency in the wild‐type plants (Figure 1H),^[^ ^16^ ^]^ but not in the knockout mutants of *GmNAS1*, *GmNAP1*, and *GmNFYC10* (Figure S5), suggesting that the GmNAS1/GmNAP1‐GmNFYC10 module attenuates the glycolysis under N‐deficiency conditions. Moreover, we observed that the expression of most of these glycolytic genes was higher in the *GmNAS1*/*GmNAP1* knockout mutant nodules than that in the wild‐type nodules under N‐deficiency conditions (Figure 1I). Considering that half of these glycolytic genes encode pyruvate kinases (PKs),^[^ ^16^ ^]^ we then examined the PK2a protein content and PK activity. The results showed that N deficiency reduced PK2a protein content and PK activity in W82 nodules, but the decrease was remarkably attenuated in the *cr‐nas1*, *cr‐nap1*, and *cr‐nas1nap1‐1* nodules (Figure S6). Accordingly, the pyruvate contents in the *cr‐nas1*, *cr‐nap1*, and *cr‐nas1nap1‐1* nodules were also greatly higher than that in the wild‐type nodules under N‐deficiency conditions (Figure 1J). The oxaloacetic acid (OAA) and malate contents in these mutant nodules were lower than those in the wild‐type nodules (Figure 1K,L), and the ratio of pyruvate to OAA was dramatically increased in the mutant nodules (Figure 1 M), suggesting that GmNAS1/GmNAP1 play an important role in regulating the competition between pyruvate and OAA production for PEP under N‐deficiency conditions.^[^ ^16^ ^]^ Taken together, these results indicated that GmNAS1/GmNAP1 inhibit GmNFYC10 nuclear accumulation to reduce pyruvate production under N‐deficiency conditions, thus enhancing OAA and malate synthesis to improve nodule NFC.

As sessile organisms, plants need to adapt to fluctuating nutritional environments to ensure their survival and optimal growth. To cope with mineral N deficiency in the soil, legumes have evolved SNF to acquire N nutrition from the atmosphere. Previous studies reported that N deficiency would promote nodule formation through inhibiting the AON pathway.^[^ ^9^, ^10^, ^11^ ^]^ Here, our study showed that N deficiency at later stages can strongly enhance nodule NFC through modulating C allocation in roots and nodules. Under N‐sufficiency conditions, more sucrose is allocated to root cells to support mineral N uptake and assimilation (Figure S7A), which is more economical than SNF.^[^ ^1^, ^2^, ^3^ ^]^ When the usable nitrogen in the soil is deficient, more sucrose would be allocated to nodules to increase NES. The elevated NES is responded by the GmNAS1/GmNAP1‐GmNFYC10 module to lower the expression of PK genes, thereby modulating PEP allocation to promote nodule NFC (Figure S7B). Therefore, under N‐deficiency conditions, C allocation in nodules is regulated by the GmNAS1/GmNAP1‐GmNFYC10 module, it will be worthy to investigate the underlying mechanism by which N deficiency regulates the C allocation in roots. Our findings reveal how legumes modulate nodule NFC to adapt to mineral N deficiency, and provide novel insights into the design of leguminous crops with improved adaptation to fluctuating usable nitrogen supplies in the soil.

## AUTHOR CONTRIBUTIONS

Xiaolong Ke and Xuelu Wang designed the experiments; Xiaolong Ke performed most of the experiments; Han Xiao contributed to the vector construction, material collection, and nodulation assays; Yaqi Peng generated the stable transgenic soybean plants; Xue Xia participated in the design of experiments; Xiaolong Ke and Xuelu Wang wrote the manuscript.

## CONFLICT OF INTEREST STATEMENT

The authors declare no conflicts of interest. Xuelu Wang is a member of the *Exploration* editorial board.

## Supporting information

Supporting Information

Supporting Information

Supporting Information

Supporting Information

Supporting Information

Supporting Information

Supporting Information

Supporting Information

## Data Availability

All data are available in the main text or supporting information. For material requests, please contact xueluw@henu.edu.cn.
