# Supplementary material for: Nitrogen deficiency modulates carbon allocation to promote nodule nitrogen fixation capacity in soybean
Source: Exploration (Beijing). 2023 Nov 30;4(2):20230104. doi: 10.1002/EXP.20230104 (PMC11022614; doi:10.1002/EXP.20230104)
Supplement: Supplementary file 1 — Supporting Information [file EXP2-4-20230104-s001.docx]

**Supporting Information**

**Nitrogen deficiency modulates carbon allocation to promote nodule nitrogen fixation capacity in soybean**

**Xiaolong Ke, Han Xiao, Yaqi Peng, Xue Xia, Xuelu Wang**

**Supplemental Experimental Procedures**

Plant materials, growth conditions and nodulation assays

Soybean (*Glycine max*) plants used in this study were in the Williams 82 background. The *cr-nas1*, *cr-nap1*, *cr-nas1nap1-1*, *cr-nas1nap1-2*, *cr-nfyc10*, and *gGmNFYC10a-FLAG* transgenic plants were created in our previous study.^[S1]^ All plants were grown at 25°C under 16-h-light/8-h-dark conditions (Photosynthetic photon flux density [PPFD] = 350 µmol m⁻² s⁻¹) and supplied with Fahraeus medium (0.5 mM MgSO_4_, 0.7 mM KH_2_PO_4_, 0.8 mM Na_2_HPO_4_, 20 µM ferric citrate, 0.68 mM CaCl_2_, 2.03 mg/L MnSO_4_, 0.08 mg/L CuSO_4_·5H_2_O, 0.22 mg/L ZnSO_4_·7H_2_O, 2.86 mg/L H_3_BO_3_, 0.08 mg/L Na_2_MoO_4_·2H_2_O, and 2 mM KNO_3_).

For nodulation assays of soybean plants, the seeds were surface sterilized with chlorine gas for 12 h and then germinated on vermiculite. The seedlings were inoculated with *B. diazoefficiens* USDA110 (OD_600_ = 0.1, 2 mL) after 8 days and supplied with Fahraeus medium. At 25 days post-inoculation (DPI), nodule number and weight were examined, and nitrogenase activity was determined by measuring acetylene reduction activity (ARA) of nodules as described previously.^[S2]^ For N-deficiency treatment, 10-DPI soybean plants were supplied with Fahraeus medium devoid of KNO_3_ for 15 days.

RT-qPCR analysis

Total RNA was extracted from various tissues using TRIpure Reagent (Aidlab), and first-strand cDNA was synthesized using M-MLV reverse transcriptase (Takara). qPCR analysis was performed with a ChamQ SYBR qPCR Master Mix (Vazyme) on a Bio-Rad CFX384 Real-Time System, and the transcript levels of the indicated genes were normalized using the expression of *TefS1*, a soybean housekeeping gene encoding the elongation factor EF-1a. The primers used for qPCR analysis are listed in our previous report.^[S1]^

Co-IP assay

For Co-IP, 25-DPI nodules of *gGmNFYC10a-FLAG* transgenic plants under N-sufficiency and N-deficiency conditions were collected and ground into a fine powder. Then, 2 mL of IP extraction buffer (50 mM Tris-HCl pH 7.5, 150 mM NaCl, 1 mM EDTA, 10% [v/v] glycerol, 1% [v/v] Triton X-100 and protease inhibitor cocktail) was added to 1 g tissue powder, and the mixture was incubated for 15 min at 4°C. After centrifugation at 4°C, 12,000 rpm for 10 min, the supernatant was incubated with ANTI-FLAG M2 Affinity Gel (Sigma) for 1 h at 4°C. The beads were washed three times with IP extraction buffer, and 1× SDS loading buffer was added to the beads. The immunoprecipitates on the beads were analyzed using immunoblotting with the corresponding antibodies, including anti-FLAG (Abmart, M20008), anti-GmNAS1 (Genscript), and anti-GmNAP1 (ABclonal). The QASSNNKKTKRNHC peptide was used to produce anti-GmNAS1 polyclonal antibody, and the SSTETSSINK peptide was used to produce anti-GmNAP1 polyclonal antibody.

Extraction of total, nuclear, and mitochondrial proteins

For the extraction of total proteins, 25-DPI nodules were collected and ground into a fine powder in liquid nitrogen. Then, 2 mL of IP extraction buffer was added to 1 g tissue powder, and the mixture was incubated for 15 min at 4°C. The supernatant was collected as total proteins after centrifugation at 4°C, 12,000 rpm for 10 min. The extraction of nuclear proteins was performed as previously described.^[S3]^ The mitochondrial purification and protein extraction of soybean nodule cells were performed mainly following the previous report.^[S4]^ Briefly, fresh nodules were first homogenized with ice-cold grinding buffer (0.4 M sorbitol, 50 mM TES buffer, 2 mM EDTA, 10 mM KH_2_PO4, 30 mM ascorbate, 2% PVP-40 and 1% BSA), and then the homogenate was filtered through 4 layers of miracloth and centrifuged at 4,000 g for 5 minutes. The supernatant was centrifuged at 10,000g for 15 min, and the pellet was resuspended with washing buffer (0.4 M sorbitol, 10 mM TES buffer pH 7.2 and 0.1% BSA). The resuspended pellet was layered over washing buffer containing 45% (v/v) Percoll, and then centrifuged at 40,000 g for 30 minutes. The tight brown band near the top of the tube was collected and layered to the top of washing buffer containing 28% (v/v) Percoll and a linear gradient of 0-10% (w/v) PVP-25, and centrifuged at 40,000g for 30 min. The purified mitochondria were found in a pale brown band near the bottom of the tube. 5% (w/v) SDS was added to the purified mitochondria and mixed for 10 min at 95°C to extract the mitochondrial proteins. Ubiquinol-cytochrome c oxidoreductase subunit CYC1 was used as the mitochondrial marker, and the CYC1 antibody (PhytoAB, PHY0566S) was used to detect the CYC1 protein. The anti-Actin (Abmart, M20009) and anti-H3 (Sigma, H0164) antibodies were used to detect the actin and histone3 proteins, respectively.

Determination of sucrose concentration

The sucrose concentration of soybean nodules, roots, and leaves was determined by a Sucrose Assay Kit (Sigma, MAK267) following the technical manual.

Determination of adenylate and organic acid contents

The ATP, ADP, AMP, pyruvate, OAA, and malate contents in soybean nodules were determined according to our previous report.^[S1]^ Energy charge (EC) was calculated with the equation EC = (ATP + 0.5 ADP)/(ATP+ADP+AMP).

Determination of pyruvate kinase activity

Pyruvate kinase (PK) activity of soybean nodules was determined by a Pyruvate Kinase Activity Assay Kit (Solarbio, China) following the technical manual. Consumption of 1 nmol NADH per minute was defined as one unit (U) of PK activity.

Accession Numbers

Sequence data of genes mentioned in this article can be found in SoyBase (<https://www.soybase.org/>) under the following accession numbers: *GmNAS1*, Glyma.06G158200; *GmNAP1*, Glyma.04G207600; *GmNFYC10a*, Glyma.12g069100; *PK2a*, Glyma.19G000700; *ACTIN*, Glyma.02G091900; *TefS1*, Glyma.17G186600.

**Supplemental References**

[S1] X. Ke, H. Xiao, Y. Peng, J. Wang, Q. Lv, X. Wang, *Science* **2022**, *378*(6623), 971.

[S2] S. Jiang, M. F. Jardinaud, J. Gao, Y. Pecrix, J. Wen, K. Mysore, *et al.*, *Science* **2021**, *374*, 625.

[S3] X. Zhao, J. Li, B. Lian, H. Gu, Y. Li, Y. Qi, *Nat. Commun.* **2018**, *9*, 5056.

[S4] D. A. Day, G. D. Price, P. M. Gresshoff, *Protoplasma* **1986**, *134***,** 121.


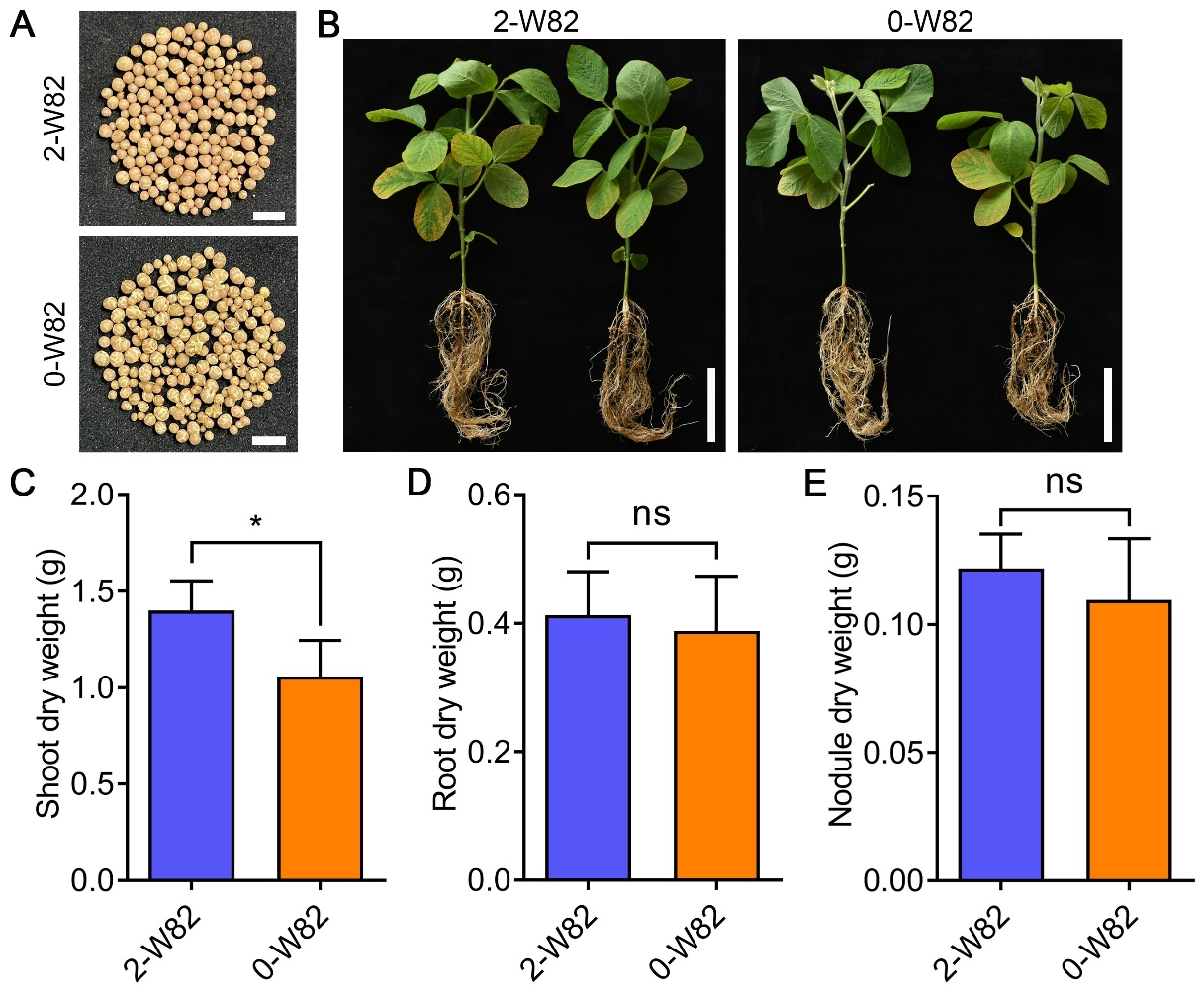


**Figure S1. The phenotype of W82 plants under** **different N conditions.**

(A and B) The nodule (A) and plant (B) phenotypes of 2-W82 and 0-W82. Scale bars, 0.5 cm in (A), and 10 cm in (B).

(C-E) Shoot (C), root (D), and nodule (E) dry weight of 2-W82 and 0-W82 plants. Data are means of four biological replicates ± SD. Significant differences were determined by Student’s *t* test (^*^*P* < 0.05); ns, not significant.


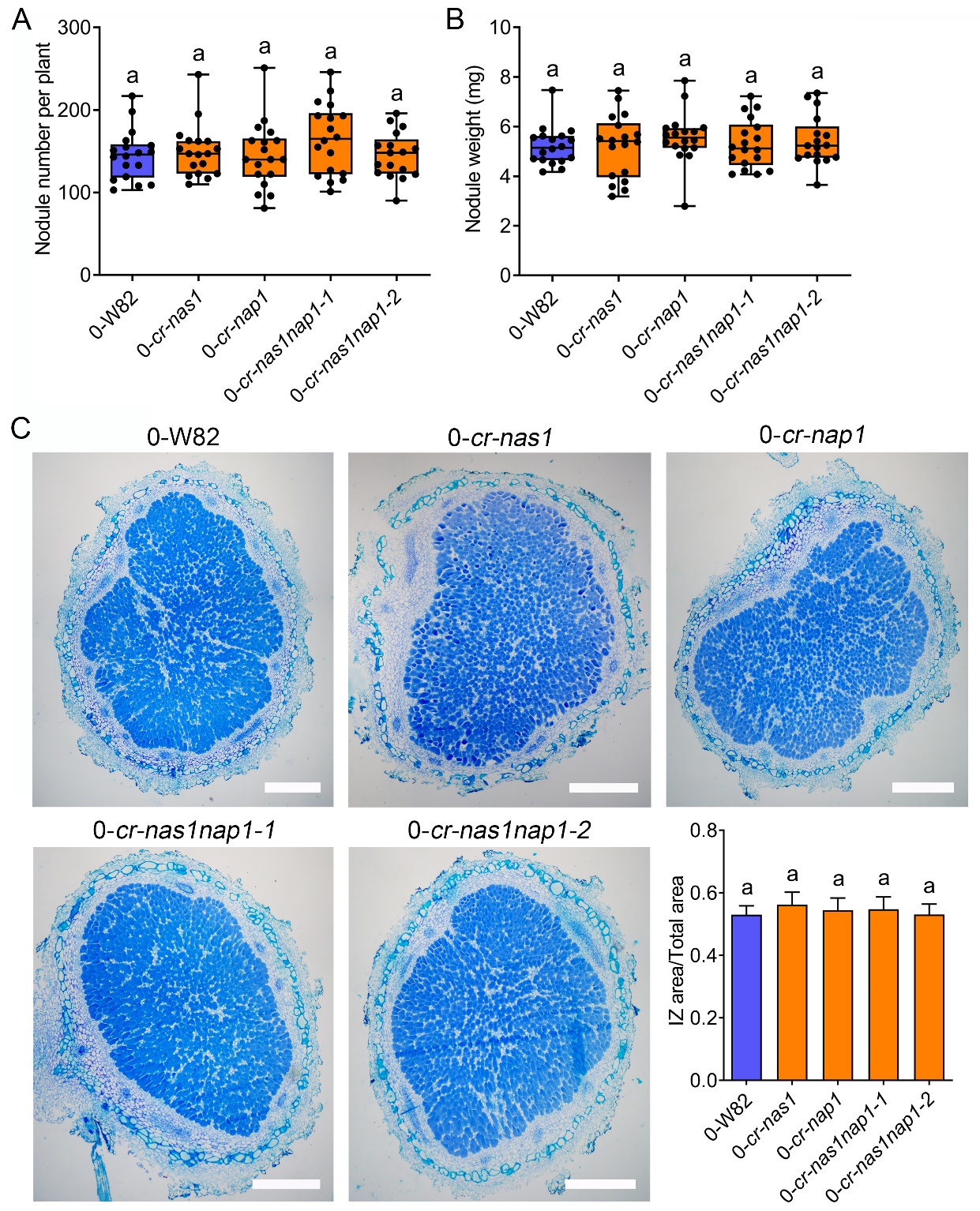


**Figure S2. The nodulation phenotype of W82 and *GmNAS1/GmNAP1* knockout mutants under N-deficiency conditions.**

(A and B) Nodule number (A) and weight (B) of W82, *cr-nas1*, *cr-nap1*, *cr-nas1nap1-1*, and *cr-nas1nap1-2* under N-deficiency conditions.

(C) Paraffin section and infection zone (IZ) observation of W82, *cr-nas1*, *cr-nap1*, *cr-nas1nap1-1*, and *cr-nas1nap1-2* nodules under N-deficiency conditions. The 25-DPI nodule slices were stained by toluidine blue. Data are means of six biological replicates ± SD.

Significant differences were determined by one-way ANOVA and post-hoc Tukey’s test with different lowercase letters indicating significant differences (*P* < 0.05).

**
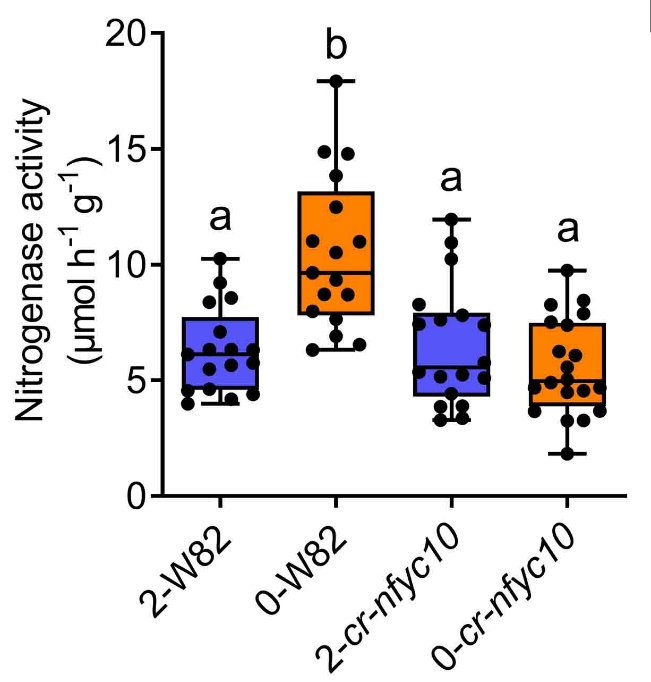
**

**Figure S3. *GmNFYC10* is needed for the increase in nodule NFC under N-deficiency conditions.**

The nodule nitrogenase activity of W82 and *cr-nfyc10* under N-sufficiency and N-deficiency conditions. The *cr-nfyc10*, knockout mutant of *GmNFYC10a* and its homolog *GmNFYC10b*.

Significant differences were determined by one-way ANOVA and post-hoc Tukey’s test with different lowercase letters indicating significant differences (*P* < 0.05).


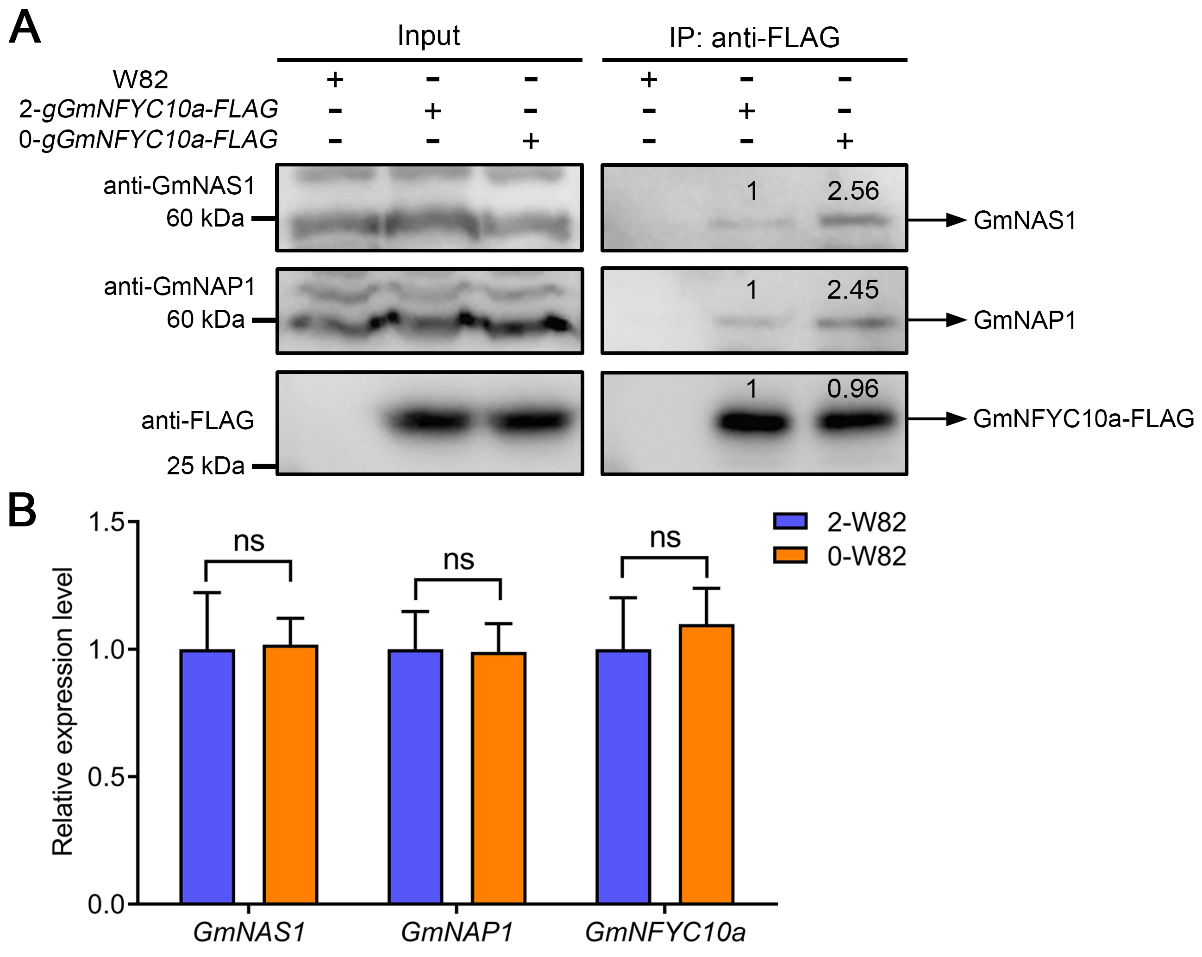


**Figure S4. N deficiency enhances the interaction of GmNAS1/GmNAP1 with GmNFYC10a in vivo.**

(A) Co-immunoprecipitation assays (Co-IP) of the interaction between GmNAS1/GmNAP1 and GmNFYC10a. The 25-DPI nodules of W82 were used as the negative control, and the 25-DPI nodules from the *gGmNFYC10a-FLAG* transgenic plants under N-sufficiency and N-deficiency conditions were used for Co-IP assays. The numbers above the lanes indicate relative band intensity quantified by ImageJ. The experiments were performed three times with comparable results.

(B) Relative expression levels of *GmNAS1*, *GmNAP1*, and *GmNFYC10a* in W82 nodules under N-sufficiency and N-deficiency conditions. Significant differences were determined by Student’s *t* test (^*^*P* < 0.05, ^**^*P* < 0.01, ^***^*P* < 0.001); ns, not significant.


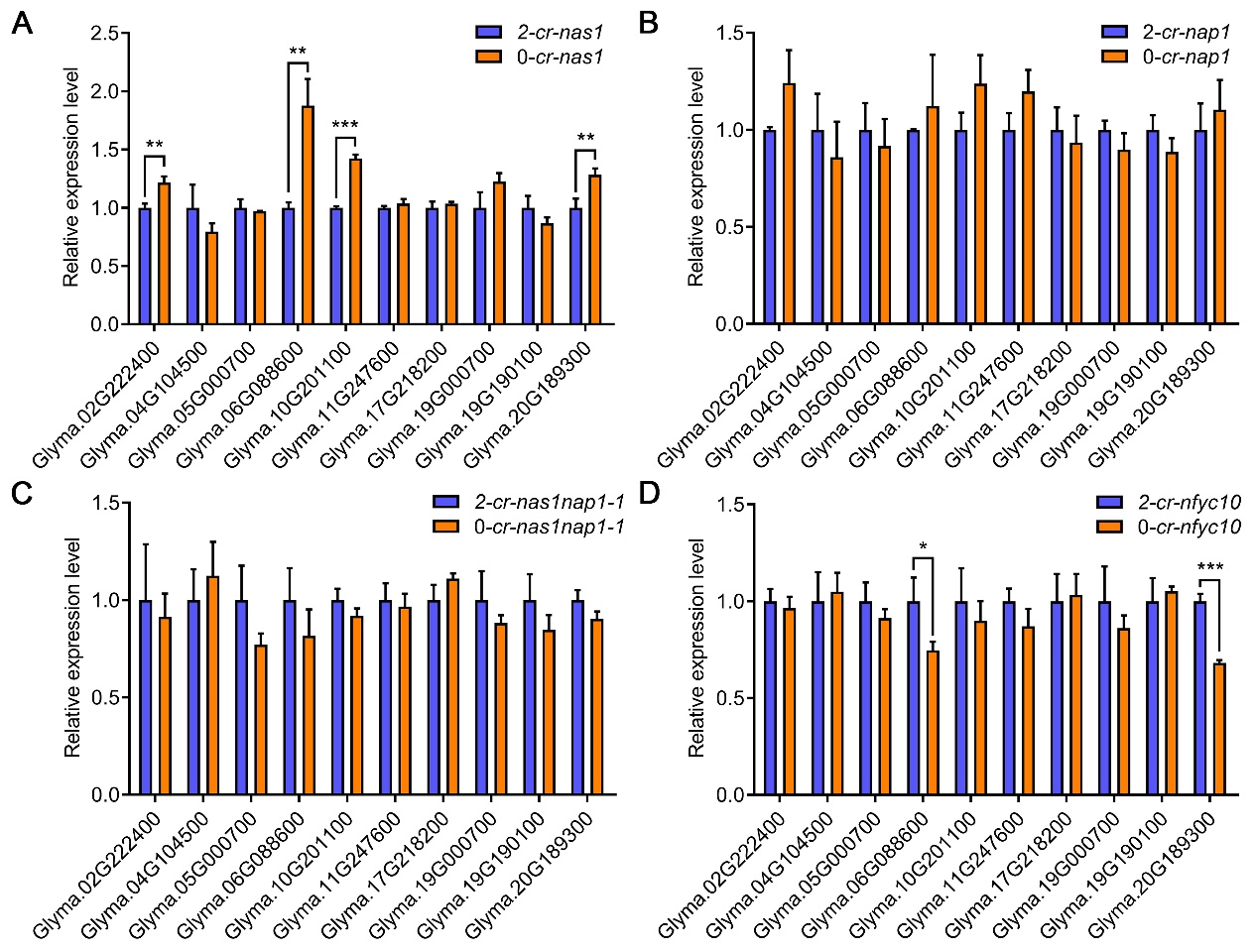


**Figure S5. The GmNAS1/GmNAP1-GmNFYC10 module regulates the downregulated expression of glycolytic genes in nodules under N-deficiency conditions.**

(A-D) Relative expression levels of glycolytic genes in *cr-nas1* (A), *cr-nap1* (B), *cr-nas1nap1-1* (C) and *cr-nfyc10* (D) nodules under N-sufficiency and N-deficiency conditions. Significant differences were determined by Student’s *t* test (^*^*P* < 0.05, ^**^*P* < 0.01, ^***^*P* < 0.001).


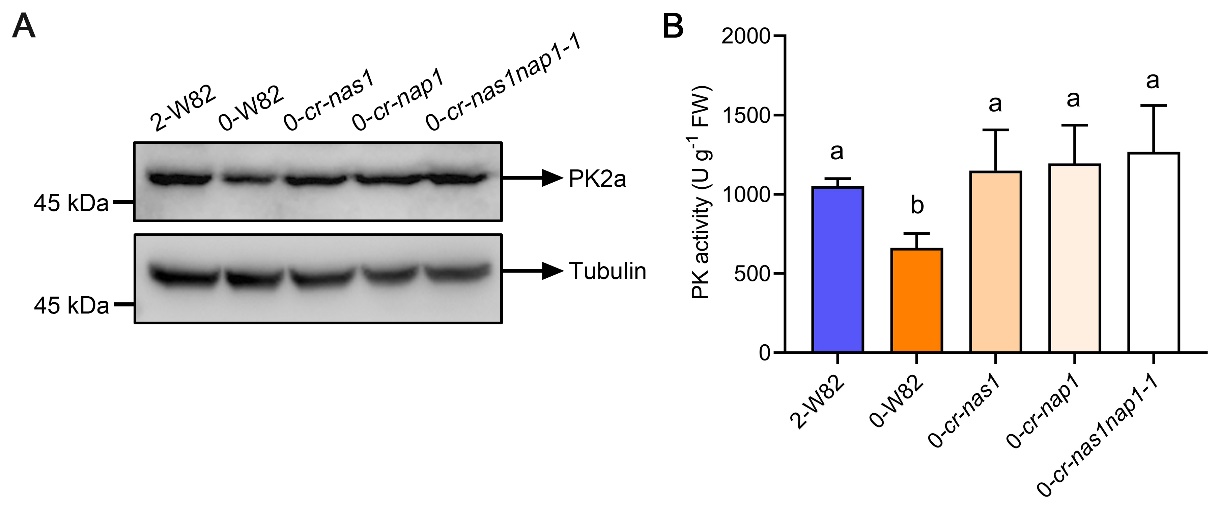


**Figure S6. GmNAS1 and GmNAP1 regulate the decrease in PK protein and activity under N-deficiency conditions.**

(A) Immunoblotting of PK2a protein levels in W82, *cr-nas1*, *cr-nap1* and *cr-nas1nap1-1* nodules after N deficiency. 2-W82 nodules were used as the control. The ITTDYDIKGDQEMI peptide was used to produce anti-PK2a polyclonal antibody (Genscript). Tubulin was used as an internal control.

(B) PK activity in W82, *cr-nas1*, *cr-nap1* and *cr-nas1nap1-1* nodules after N deficiency. 2-W82 nodules were used as the control. Data are means of four biological replicates ± SD. Significant differences were determined by one-way ANOVA and post-hoc Tukey’s test with different lowercase letters indicating significant differences (*P* < 0.05).


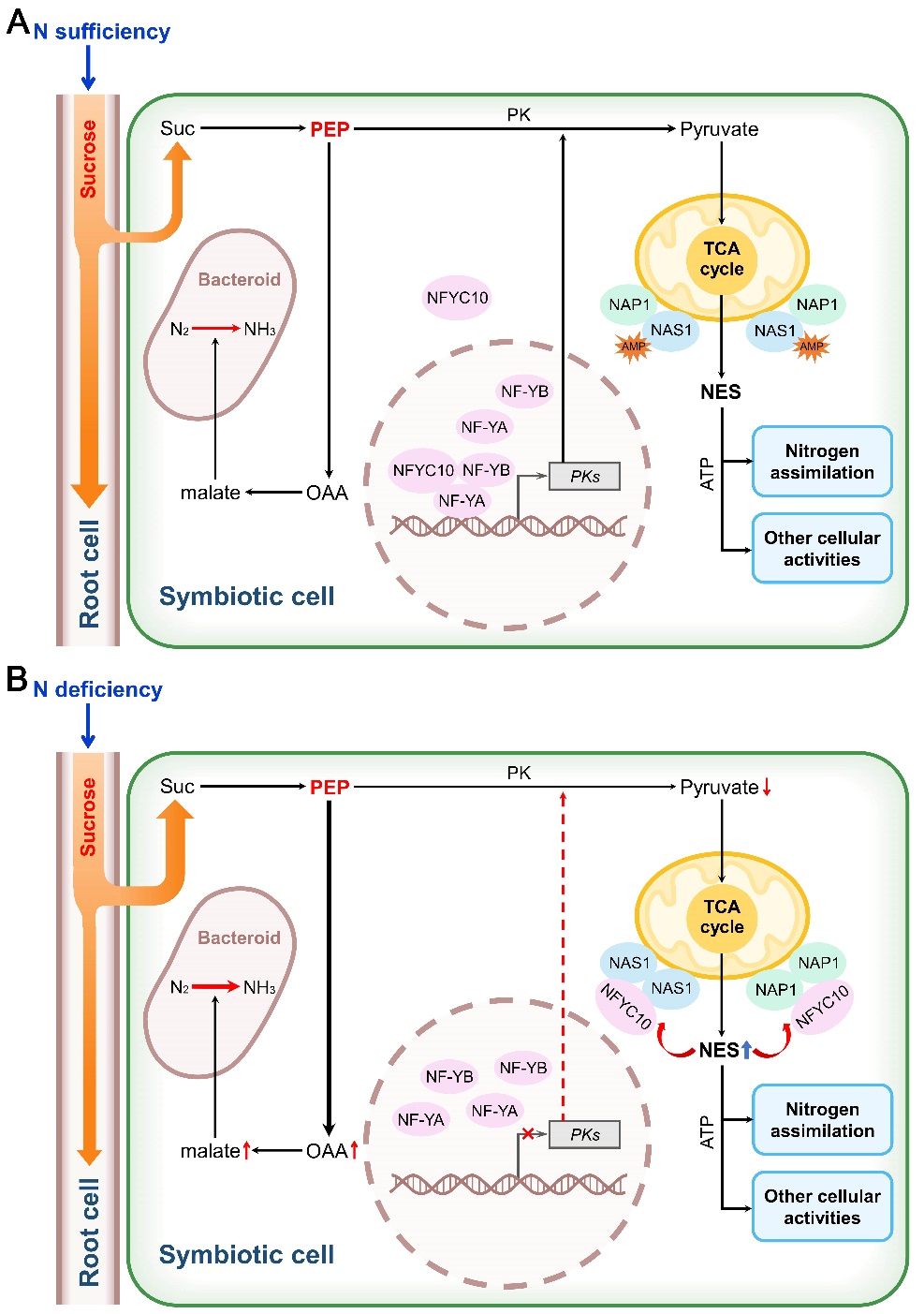


**Figure S7. The model of** **GmNAS1 and GmNAP1 modulating nodule NFC under different N conditions.**

(A) Schematic diagram of soybean root and nodule responding to N sufficiency.

(B) Schematic diagram of soybean root and nodule responding to N deficiency.
