# Supplementary figures and images for: Nitrogen deficiency modulates carbon allocation to promote nodule nitrogen fixation capacity in soybean
Source: Exploration (Beijing). 2023 Nov 30;4(2):20230104. doi: 10.1002/EXP.20230104 (PMC11022614; doi:10.1002/EXP.20230104)

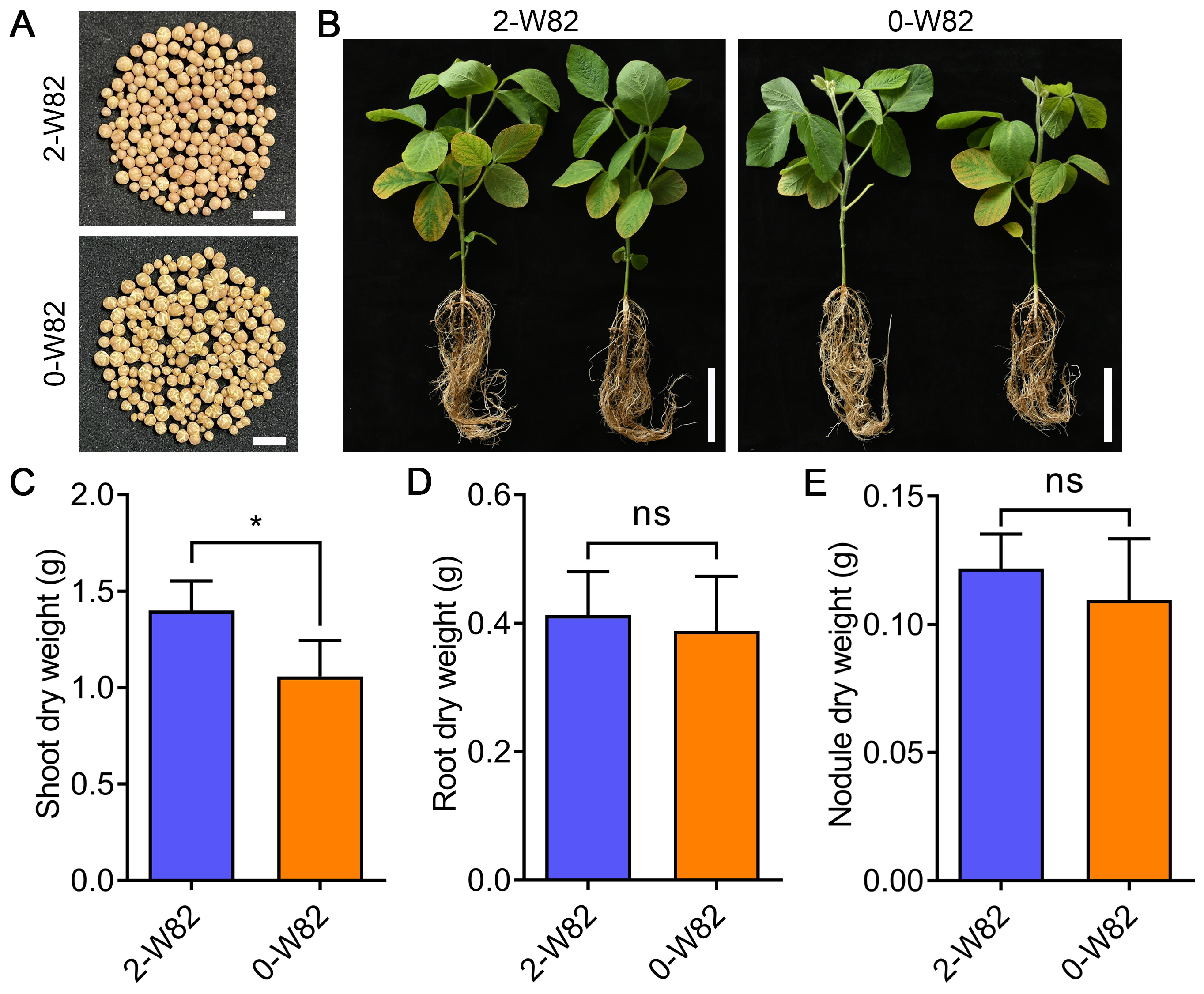

Supplement: Supplementary file 2 — Supporting Information [file EXP2-4-20230104-s008.tif]

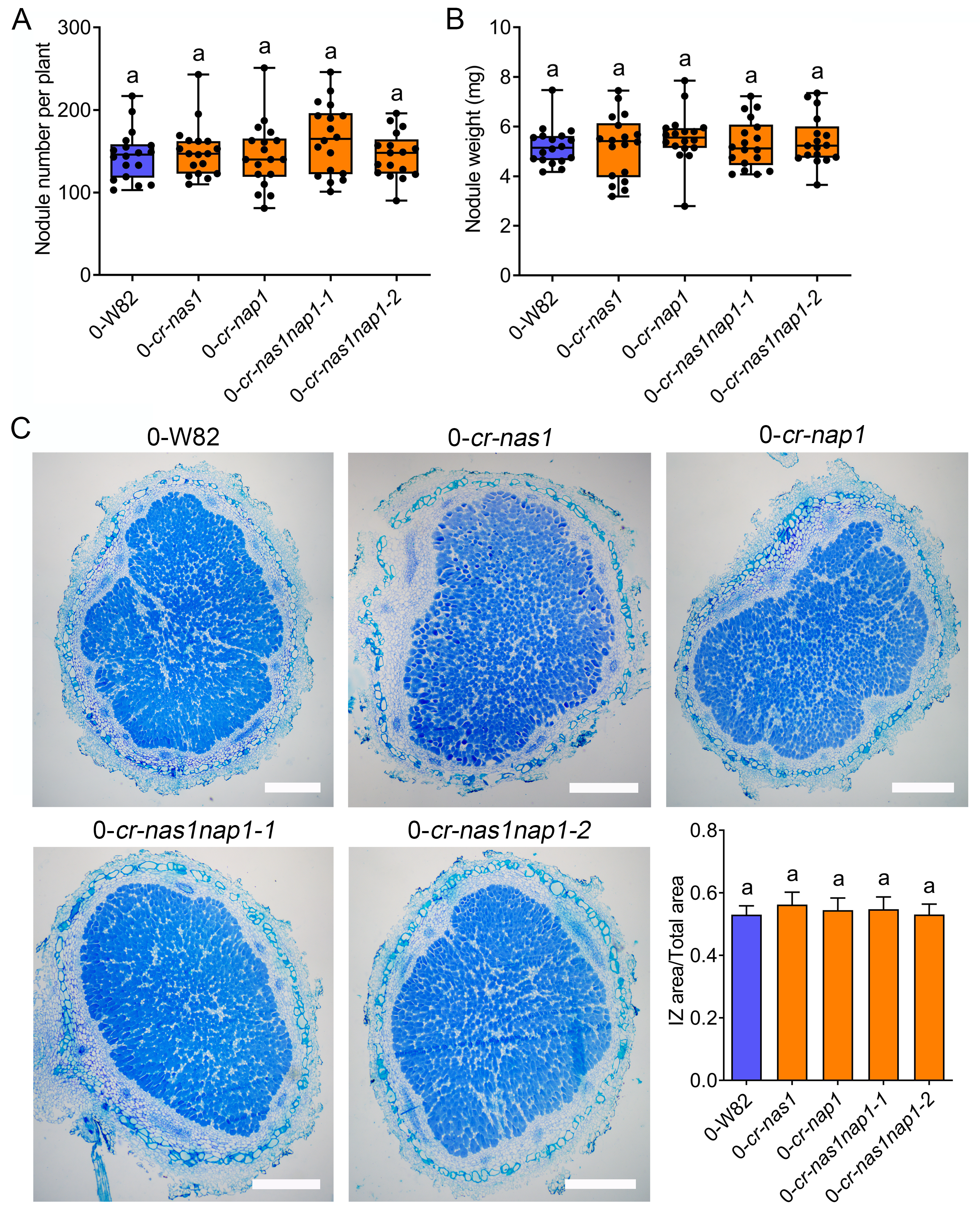

Supplement: Supplementary file 3 — Supporting Information [file EXP2-4-20230104-s006.tif]

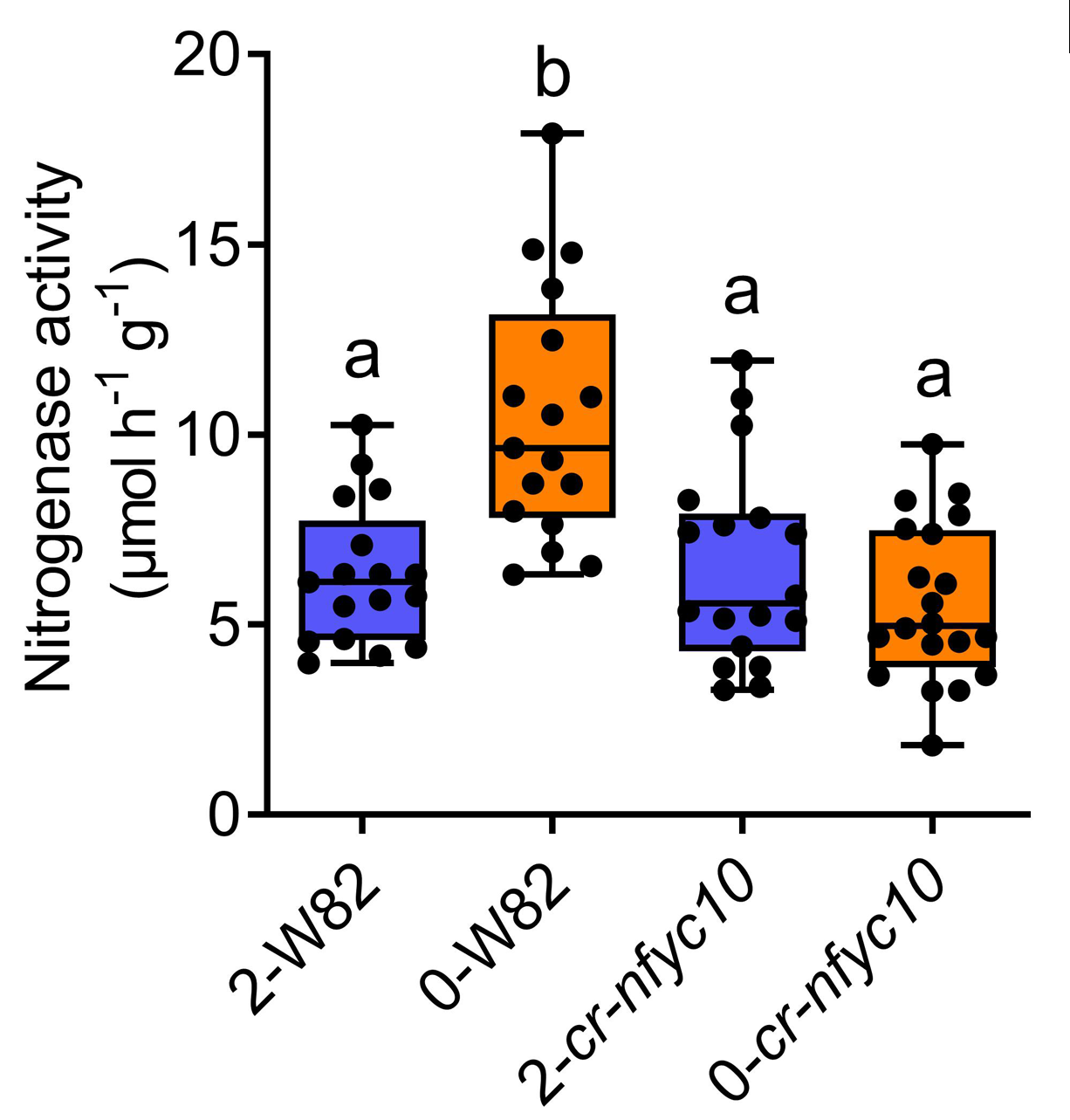

Supplement: Supplementary file 4 — Supporting Information [file EXP2-4-20230104-s002.tif]

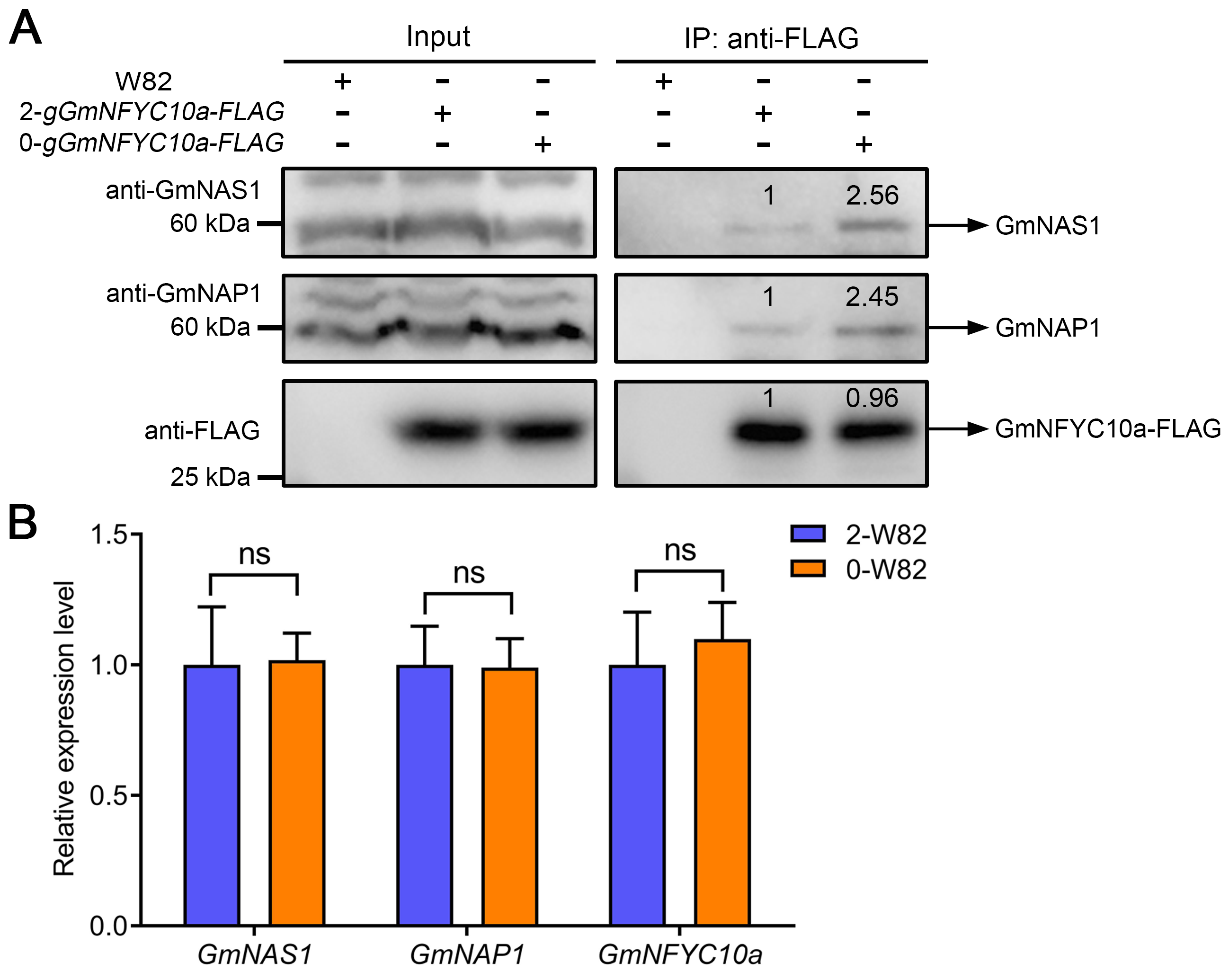

Supplement: Supplementary file 5 — Supporting Information [file EXP2-4-20230104-s005.tif]

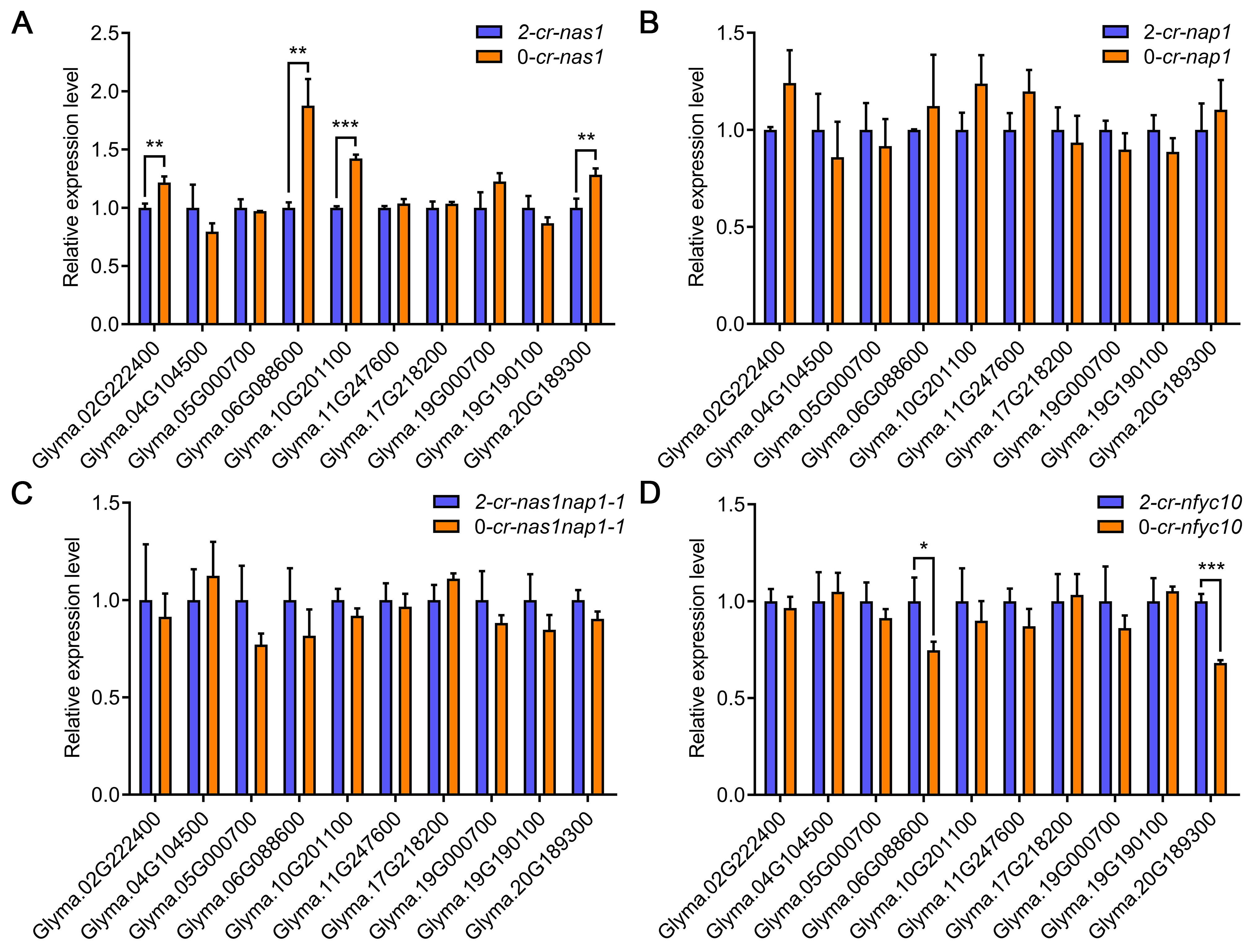

Supplement: Supplementary file 6 — Supporting Information [file EXP2-4-20230104-s003.tif]

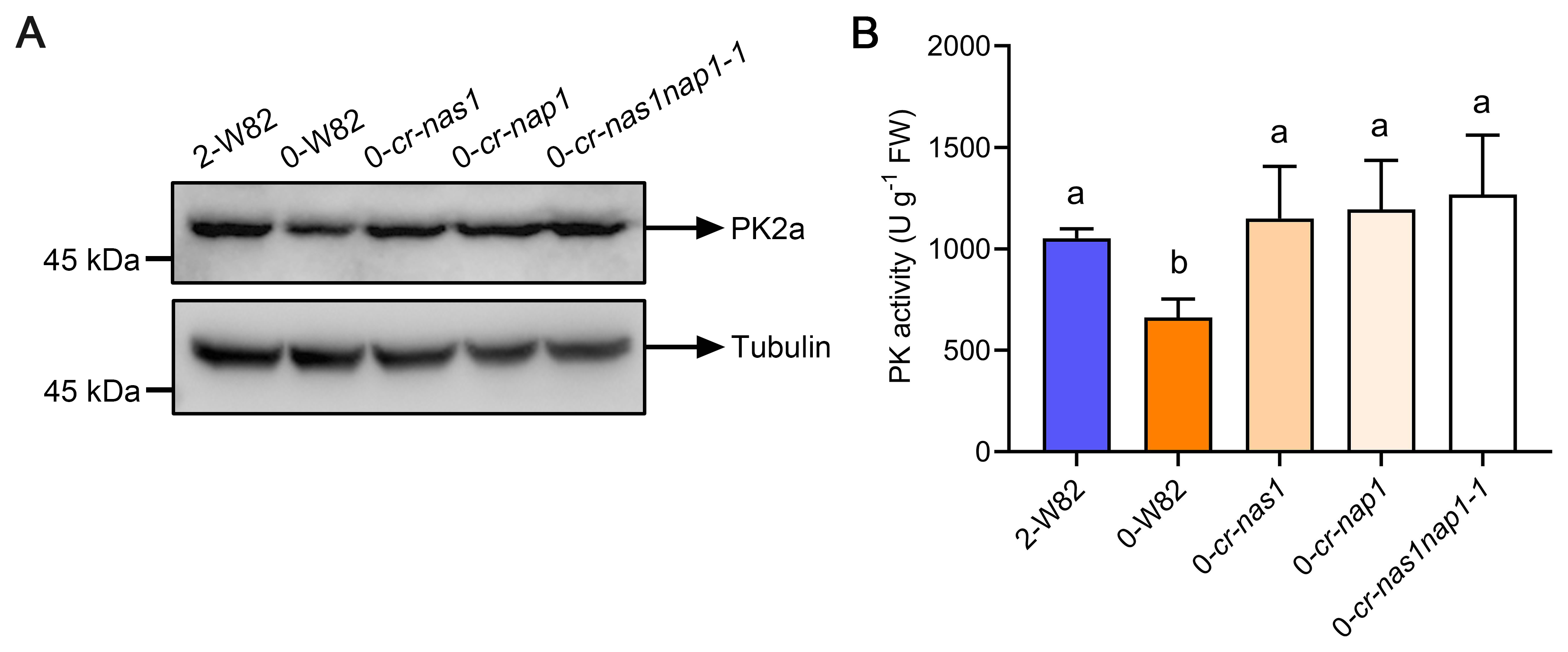

Supplement: Supplementary file 7 — Supporting Information [file EXP2-4-20230104-s004.tif]
